# Sex differences in laterality of motor unit firing behavior of the first dorsal interosseous muscle in strength-matched healthy young males and females

**DOI:** 10.1007/s00421-024-05420-7

**Published:** 2024-02-16

**Authors:** Yuichi Nishikawa, Kohei Watanabe, Aleš Holobar, Ryoka Kitamura, Noriaki Maeda, Allison S. Hyngstrom

**Affiliations:** 1 Faculty of Frontier Engineering, Institute of Science & Engineering, Kanazawa University, Kakuma-machi, Kanazawa, 920-1192 Japan; 2 Laboratory of Neuromuscular Biomechanics, School of Health and Sport Sciences, Chukyo University, Nagoya, Japan; 3 Faculty of Electrical Engineering and Computer Science, University of Maribor, Maribor, Slovenia; 4 Graduate School of Frontier Engineering, Kanazawa University, Kanazawa, Japan; 5 Division of Sports Rehabilitation, Graduate School of Biomedical and Health Sciences, Hiroshima University, Hiroshima, Japan; 6 Department of Physical Therapy, Marquette University, Milwaukee, WI USA

**Keywords:** Sex difference, Motor unit, Dominant hand, First dorsal interosseous muscle

## Abstract

**Purpose:**

The purpose of this study was to compare laterality in motor unit firing behavior between females and males.

**Methods:**

Twenty-seven subjects (14 females) were recruited for this study. The participants performed ramp up and hold isometric index finger abduction at 10, 30, and 60% of their maximum voluntary contraction (MVC). High-density surface electromyography (HD-sEMG) signals were recorded in the first dorsal interosseous (FDI) muscle and decomposed into individual motor unit (MU) firing behavior using a convolution blind source separation method.

**Results:**

In total, 769 MUs were detected (females, n = 318 and males, n = 451). Females had a significantly higher discharge rate than males at each relative torque level (10%: male dominant hand, 13.4 ± 2.7 pps vs. female dominant hand, 16.3 ± 3.4 pps; 30%: male dominant hand, 16.1 ± 3.9 pps vs. female dominant hand, 20.0 ± 5.0 pps; and 60%: male dominant hand, 19.3 ± 3.8 vs. female dominant hand, 25.3 ± 4.8 pps; *p* < 0.0001). The recruitment threshold was also significantly higher in females than in males at 30 and 60% MVC. Furthermore, males exhibited asymmetrical discharge rates at 30 and 60% MVC and recruitment thresholds at 30 and 60% MVC, whereas no asymmetry was observed in females.

**Conclusion:**

In the FDI muscle, compared to males, females exhibited different neuromuscular strategies with higher discharge rates and recruitment thresholds and no asymmetrical MU firing behavior. Notably, the findings that sex differences in neuromuscular activity also occur in healthy individuals provide important information for understanding the pathogenesis of various diseases.

## Introduction

Sex differences in physical performance have been well documented across a variety of activities (Lewis et al. 1986; Conkright et al. 2022). The mechanisms of sex differences are multifactorial, and differences in strength, muscle volume and muscle fiber composition (Bishop et al. 1987; Staron et al. 2000) have been documented. Less information is known about sex differences in the central nervous system that underlie force regulation (Nishikawa et al. 2017a; Inglis and Gabriel 2020; Taylor et al. 2022). Understanding sex differences between male and female motor unit (MU) firing behavior is important because knowledge of sex-specific neuromuscular control improves health and well-being by providing insights into aging, disease, and training.

A recent review reported that several muscles (vastus lateralis (VL), vastus medialis (VM), tibialis anterior (TA), and first dorsal interosseous (FDI) muscles) exhibit different MU firing behavior between females and males on intramuscular electromyography (EMG) and high-density surface EMG (HD-sEMG) (Lulic-Kuryllo and Inglis 2022). A common sex difference identified in these muscles was that females exhibited a higher discharge rate of MUs than males (Peng et al. 2018; Parra et al. 2020; Inglis and Gabriel 2020; Guo et al. 2022). Specifically, females have shown a higher discharge rate of MUs at lower intensities (10–40% MVC) for the TA (Inglis and Gabriel 2020) and VL (Guo et al. 2022) muscles but a higher discharge rate of MUs at higher intensities (> 60% MVC) for VM (Peng et al. 2018) and FDI (Parra et al. 2020) muscles during isometric contraction compared to males. These findings suggested that there are potential sex-related differences in neural drive that contribute to force output.

Another important factor that may influence sex differences in MU firing behavior is the difference in maximal muscle strength between females and males (Boccia et al. 2019). In several studies that have examined sex differences in MUs, participants with sex-related differences in maximal muscle strength have been recruited (Nishikawa et al. 2017a; Parra et al. 2020; Inglis and Gabriel 2020; Taylor et al. 2022). Musculoskeletal differences between females and males may play an important role in sex differences in MU firing behavior (Oliveira et al. 2022). Therefore, it is important to match females and males by maximal muscle strength. A recent study reported that sex differences in the properties of MUs in the TA muscle detected using intramuscular EMG were more apparent when the MVC values were matched (Inglis and Gabriel 2020). However, no study has been conducted to support a sex difference in MUs in the FDI muscle in strength-matched subjects.

Dominance is another known factor that influences MU firing behavior, especially in the upper extremities (Adam et al. 1998). Using the intramuscular EMG method, Adam et al. reported that the dominant FDI muscle exhibited lower discharge rates and recruitment thresholds than the nondominant FDI muscle during 30% submaximal isometric contraction in males (Adam et al. 1998). This finding may be influenced by differences in adaptations of the muscle in response to preferential use. The lateral dominance of the cerebral cortex indicates functional specialization within the left or right cerebral hemisphere of the brain. An essential principle of human brain organization is functional cerebral asymmetry (FCA), which is thought to result from interhemispheric inhibition of the dominant hemisphere. It has been reported that FCA is sex specific: males have more stable FCA (greater asymmetry) than females (Weis and Hausmann 2010).

The aim of this study was to examine the sex and laterality differences in MU firing behavior during submaximal isometric contractions of the FDI muscle in strength-matched healthy young males and females using HD-sEMG method. We hypothesized that compared to males, females would exhibit higher MU discharge rates and lower dominant vs. nondominant hand asymmetry of MU firing behavior. HD-sEMG is a noninvasive method for assessing the behavior of MUs and can be applied to a wide range of subjects. Recently, its accuracy for tracking MUs was reported (Goodlich et al. 2023). Given the high applicability of this method, including the ability to track activity changes in MUs over time, we believe that identifying sex differences in the FDI muscle, which is commonly used in the assessment of several neurodegenerative diseases, is important.

## Materials and methods

### Participants

Twenty-seven subjects (females, n = 14 and males, n = 13) were enrolled in this study after written informed consent was obtained (Table 1). The inclusion criteria were independence in activities of daily life and the ability to give informed consent. The exclusion criteria were a reported history of orthopedic, neuromuscular, and cardiovascular diseases or diabetes mellitus. This study was approved by the Research Ethics Committee of Kanazawa University (approval no. 2020-220 (83)) and was performed in accordance with the Declaration of Helsinki.

**Table 1:** Characteristics of participants

	Males, n = 13	Females, n = 14
Age, years	22.4 ± 1.0	22.2 ± 0.9
BMI, kg/m^2^	20.7 ± 2.0	19.8 ± 1.5
Subcutaneous tissue, mm Dominant side/Nondominant side	2.65 ± 0.72/2.54 ± 0.45	2.88 ± 0.65/3.30 ± 0.79
Cross sectional area of first dorsal interosseous muscle, cm^2^ Dominant side/Nondominant side	2.80 ± 0.97/2.34 ± 0.44	1.95 ± 0.40/1.79 ± 0.59
MVC, N Dominant side/Nondominant side	47.7 ± 26.1/44.8 ± 23.9	43.3 ± 22.9/39.6 ± 21.1
Dominant side	Right: 11, Left: 2	Right: 12, Left: 2

### Experimental procedures

### Measurements

A total of two visits to the laboratory were made by participants. As part of the first visit, the participants were introduced to the experimental procedures by performing a series of maximal and submaximal isometric finger abductions. Participants underwent the main experimental session during the second visit, which occurred 24 h after the familiarization session. During this examination, ultrasound was used (to assess muscle cross-sectional area (CSA) and thickness of subcutaneous tissue) as well as to record voluntary isometric finger abduction force and HD-sEMG signals from FDI muscle.

Maximum voluntary contraction (MVC).

**Fig. 1:**
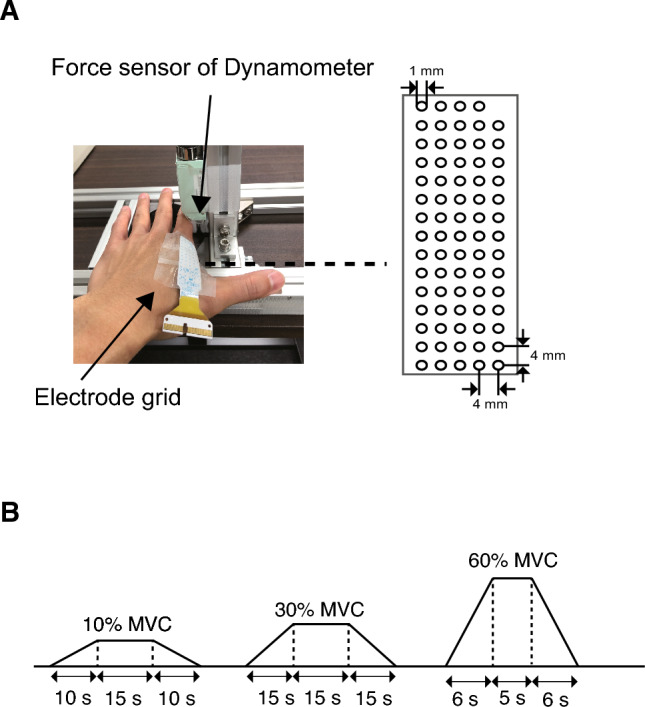
Placement of the electrode grid and study protocol. **A** An electrode grid was placed on the FDI muscle belly. The force sensor of the dynamometer was placed to touch the outside of the basal phalanx of the index finger. **B** Participants performed three submaximal voluntary contractions

After placement of the surface electrodes, the participants were asked to perform finger abduction at MVC, and the finger testing order was randomized. The subject’s hand rested palm down on the examination table with the thumb in a 90-degree radial abduction position. A dynamometer (Takei Scientific Instruments Co., Ltd., Niigata, Japan) was used to measure the MVC (Fig. 1B). The force signal was detected by a force amplifier (TSA-110, Takei Scientific Instruments Co., Ltd., Niigata, Japan) with a 190 Hz sampling rate. The subjects were instructed to maintain a sitting posture during the MVC measurement. All participants performed two MVC trials after a warm-up period of ten minutes that included upper limb stretching and indoor walking. The target torque for the submaximal isometric pinch ramp-up contractions was calculated from the peak MVC torque. During MVC measurements, the subject was asked to keep the upper arm in a 90-degree flexed position at the shoulder and elbow joints. The subject’s palms were placed on the table to prevent wrist and finger flexion.

### Ultrasound

Ultrasound images of the FDI muscle were taken bilaterally to determine the muscle cross-sectional area (CSA) and thickness of subcutaneous tissue using an ultrasound imaging device (FAMUBO, SEIKOSHA, Tokyo, Japan). During the examination, participants sat in the same chair as for MVCs with the tested hand open. For each scan, a 2-cm scan depth was used, and the transducer frequency was 7.5 MHz for ultrasound brightness mode (B-mode). A longitudinal scan of the muscle was used to identify the FDI's origin and insertion. We measured and marked the origin and insertion. A CSA measurement was conducted at the midway point between the two. The probe head was oriented perpendicular to the second metacarpal once the midway point was determined. The FDI runs along the lateral side of the second metacarpal, which was used to guide the orientation of the probe. To create uniform pressure on the skin, we ensured that enough gel was used and that the probe was perpendicular to the surface. After properly focusing the muscle, an image was captured and saved. For subsequent analysis, each image was saved in jpg format and exported to a personal computer. ImageJ (National Institutes of Health, Bethesda, MD, USA) was used to determine the muscle CSA (in cm^2^) and thickness of subcutaneous tissue (in mm). A centimeter mark was inlaid in each image to calibrate the scale. Using the muscle's cross-sectional center as a reference point, the thickness of subcutaneous tissue was measured. The CSA and polygonal tools were used to outline the entire muscle.

### HD-EMG

A grid of 64 electrodes (GR04MM1305, OT Bioelettronica) was used to record HD-sEMG signals from the FDI muscle (diameter of 1 mm, distance of 4 mm between the electrodes; Fig. 1B). Using a bioadhesive foam (KIT04MM1305, OT Bioelettronica) and conductive paste (Elefix ZV-181E, NIHON KOHDEN, Tokyo, Japan), the electrode grid was attached to the muscle surface (Nishikawa et al. 2017b, 2018). An electrode was placed at the wrist as a reference. We recorded monopolar HD-sEMG signals using an analog-to-digital converter (muovi + Pro, OT Bioelettronica, sampling frequency 2,000 Hz). A gain of 205 was applied to the signals, and they were off-line bandpass filtered between 10 and 500 Hz. Analysis of force and HD-sEMG signals was performed using MATLAB software (MATLAB 2022b, Math Works GK, MA, USA).

### Protocol

First, participants were evaluated using ultrasound for muscle shape and subcutaneous tissue thickness. Second, the electrode grid was placed to the muscle belly of the FDI muscle, after which the MVC was measured. After recording the MVC measurements, all participants performed a submaximal isometric finger abduction force at 10, 30, and 60% MVC in a random order (Fig. 1B). To calculate the discharge rate and recruitment threshold of MUs to understand MU firing behavior, a submaximal isometric contraction task was performed, for which a trapezoidal motor task was chosen based on the methods applied in previous studies (Nishikawa et al. 2017b, 2018, 2022). Contractions at 10 and 30% were sustained for 15 s, whereas those at 60% MVC lasted for 5 s. In each trial, the subjects received visual feedback of the torque applied to the dynamometer, which was displayed as a trapezoid (ramp up and ramp down): increasing torque by 1% MVC/s until 10% MVC, which was maintained for 15 s (Nishikawa et al. 2018), increasing torque by 2% MVC/s until 30% MVC, which was maintained for 15 s (Nishikawa et al. 2022), and increasing torque by 10% MVC/s until 60% MVC, which was maintained for 5 s (Nishikawa et al. 2017b). HD-sEMG data were collected during the MVC assessment and during submaximal ramp-up contraction tasks.

### Data processing

**Fig. 2:**
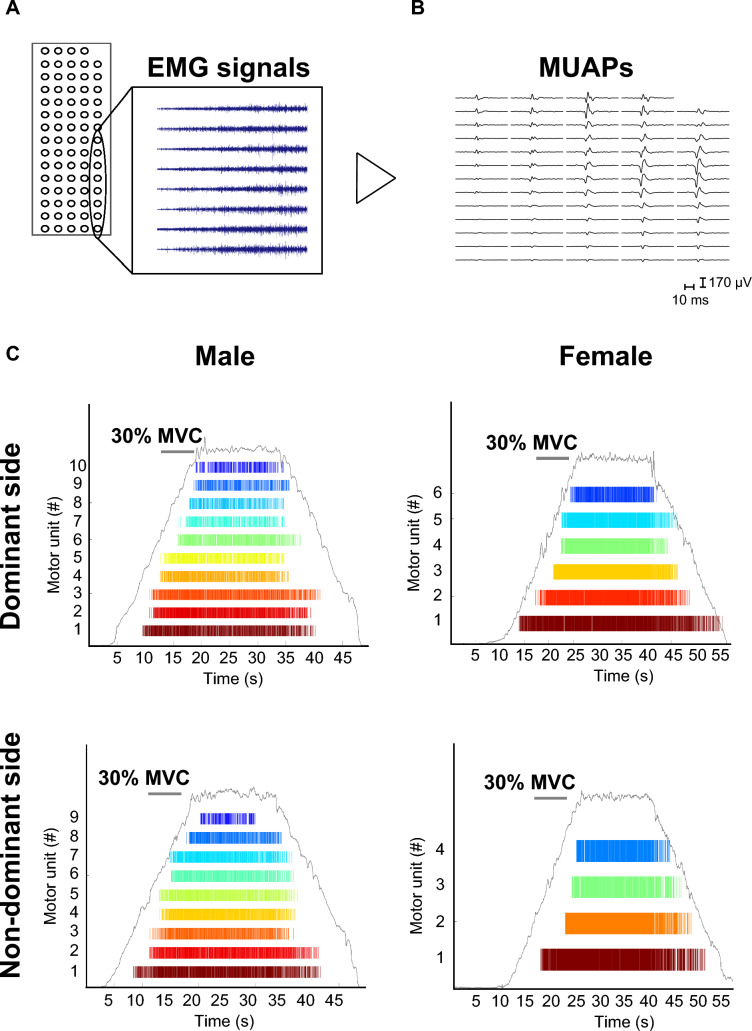
Representative images of high-density surface electromyogram (EMG) decomposition and definition of the recruitment threshold. **A** HD-sEMG signal for the 8 channels. **B** Motor unit action potentials (MUAPs) were identified by HD-sEMG decomposition. **C** Representative images of HD-sEMG decomposition in males (left side) and females (right side) (upper panel is dominant side, lower panel is nondominant side)

In this study, 59 bipolar EMG signals were calculated from adjacent electrodes. Convolutive blind source separation was used to separate HD-sEMG recordings into individual MU discharges (Holobar and Zazula 2007; Holobar et al. 2009; Holobar and Farina 2014) (Fig. 2). To identify individual MUs, we used DEMUSE software (v. 6.0; the University of Maribor, Slovenia). Data were excluded from the analysis if the discharge rate fell below 4 Hz (Holobar et al. 2009; Nishikawa et al. 2022) or the pulse-to-noise ratio was less than 30 dB (Holobar et al. 2014). The coefficient of variation (CV) for the interspike interval was defined as the ratio between the standard deviation and the mean value of the interspike interval. Next, the mean discharge rate of the identified MUs was calculated during the sustained contractions (Fig. 1B). The MU recruitment thresholds were defined by the level of force (%MVC) expected at the first firing of each MU. A wide range of MUs were recruited in the 60% MVC task. The characteristics of MUs include a phenomenon called “onion skin”, in which earlier recruited MUs generally have a higher discharge rate than that of later recruited MUs (De Luca and Hostage 2010), and a phenomenon called “reverse onion skin”, in which later recruited MUs have a higher discharge rate than that of earlier recruited MUs (Inglis and Gabriel 2021a). According to these characteristics of MUs, we classified the detected MUs into three subgroups for each RT (MU20, < 20% MVC; MU40, 20–40% MVC; MU60, 40% <). In addition, the CV of force (standard deviation (SD)/mean 100, CV force) at each level of sustained submaximal contraction was calculated.

### Statistical analysis

Stata version 17 (Stata Corp LLC, Texas, USA) was used for all analyses, while GraphPad Prism version 8 (GraphPad Software Inc., California, USA) was used to generate graphs. Data normality was confirmed using the Shapiro‒Wilk test. Based on the normality in the data, parametric analysis was performed to compare age, height, and weight in each participant between females and males using an unpaired *t* test. Furthermore, the MVC value was analyzed using two-way (sex (female and male) and side (dominant and nondominant)) analysis of variance (ANOVA). Nonparametric analysis (generalized linear mixed-effects model with random slopes) was performed to compare CSA, subcutaneous tissue, CV of force, mean discharge rate and recruitment threshold. The explanatory variables were as follows: for CSA and subcutaneous tissue, sex (female and male) and side (dominant and nondominant); for discharge rate and recruitment threshold, sex (female and male), side (dominant and nondominant), and contraction level (10, 30, and 60% of MVC). Furthermore, an analysis of the mean discharge rate at the 60% MVC task was conducted using generalized linear mixed-effects with a random intercept and a random slope. There were two explanatory variables: the side (dominant and nondominant) and MU_subgroup (MU20, MU40, and MU60). For multiple comparisons, the Bonferroni correction was applied to account for the effects of multiple comparisons. A bivariate correlation analysis was conducted using Spearman’s correlation coefficients to assess bivariate correlations between subcutaneous tissue thickness and the yield of MUs and between CSA and MVC. Effect size was calculated from the generalized linear mixed-effects model and two-way ANOVA as partial eta squared. The significance level was set at *p* < 0.05.

## Results

The general characteristics of the participants are presented in Table 1. Males and females were similar in age and body mass index (*p* = 0.6895, 95% CI =  − 0.169 to 0.670 years and *p* = 0.2192, 95% CI =  − 2.432 to 0.591 kg/m^2^).

We identified a total of 769 MUs (females: 318 MUs (dominant side = 162, nondominant side = 156); males: 451 MUs (dominant side = 242, nondominant side = 209)) that were considered for further analysis (Table 2). Furthermore, there was no correlation between subcutaneous tissue thickness and the yield of MUs in either females (*r* = − 0.2326, *p* = 0.2336) or males (*r* = − 0.09844, *p* = 0.6797).

**Table 2:** Motor Unit yield

	Males	Females
10% MVC, Dominant/Nondominant	96/89	66/82
30% MVC, Dominant/Nondominant	76/62	43/46
60% MVC, Dominant/Nondominant	70/58	53/28

**Fig. 3:**
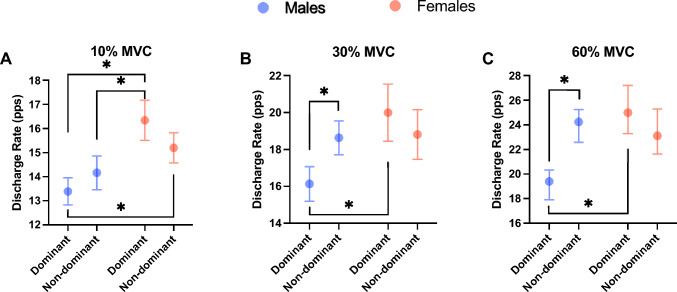
Comparison of the discharge rate between the dominant side and nondominant side in females and males at 10% (**A**), 30% (**B**), and 60% MVC (**C**). Females’ dominant side showed a significantly higher discharge rate than males’ dominant side at 10, 30, and 60% MVC. Females’ nondominant side showed a significantly higher discharge rate than the males’ dominant side at 10% MVC. Furthermore, the males’ dominant side showed a significantly higher discharge rate than the males’ nondominant side at 30 and 60% MVC. Data are shown as the median and 95% CI. * *p* < 0.05.

The discharge rate showed a significant sex $\times$ side $\times$ MVC interaction (*F* = 4.28, *p* = 0.0139, *η*^2^ = 0.060). The discharge rate showed significantly higher values at each relative torque level on the female dominant side than on the male dominant side (10% MVC: *p* < 0.0001, 95% CI =  − 4.259 to − 1.644 pps, 30% MVC: *p* < 0.0001, 95% CI =  − 5.714 to − 2.020 pps, 60% MVC: *p* < 0.0001, 95% CI =  − 8.115 to − 3.933 pps) (Fig. 3). At 10% MVC, males’ nondominant side showed a significantly lower discharge rate than females’ dominant side (*p* = 0.001, 95% CI =  − 3.503 to 0.851 pps), and males’ dominant side showed a significantly lower discharge rate than females’ nondominant side (*p* = 0.0007 and 95% CI =  − 3.036 to − 0.5857) (Fig. 3A). At 30% and 60% MVC, males’ nondominant side showed a significantly higher discharge rate than males’ dominant side (*p* = 0.0055, 95% CI =  − 4.477 to − 0.526 pps and *p* < 0.0001, 95% CI =  − 6.543 to − 2.464 pps), but this pattern was not found in females (30% MVC; *p* = 0.7068, 95% CI =  − 1.179 to 3.544 pps and 60% MVC; *p* = 0.2047 95% CI =  − 0.5624 to 4.752 pps). In contrast, at 10% MVC, male and female subjects did not show significant differences between the dominant and nondominant sides.

**Fig. 4:**
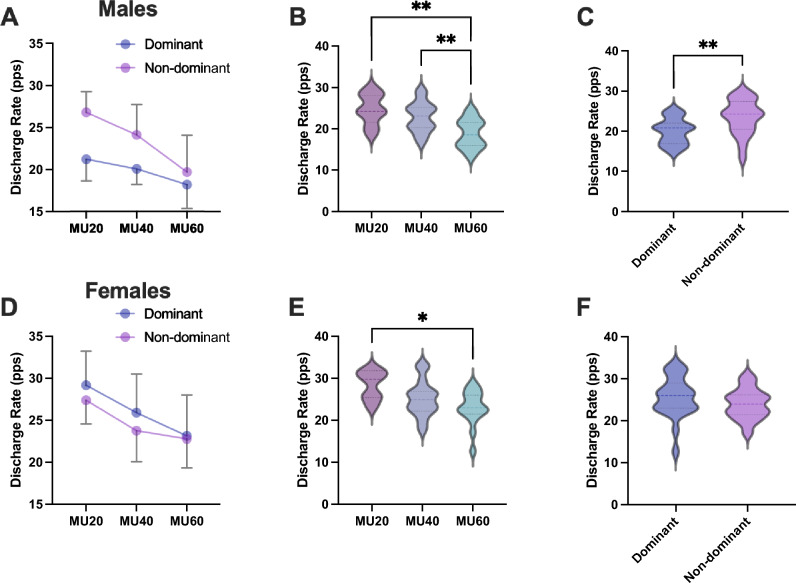
Comparison of the discharge rate of the 60% maximum voluntary contraction task between the side and MU_subgroup in males (**A**–**C**) and females (**D**–**F**). There were no significant side $\times$ MU_subgroup interactions in males and females (**A** and **D**). Males and females (**B** and **E**) had a main effect of MU_subgroup, with MU20 and MU40 discharge rates significantly higher than those of MU60 for males, while MU20 discharge rates were significantly higher than those of MU60 for females. Males’ nondominant side showed a significantly higher discharge rate than males’ dominant side (**C**), while there was no significant difference between the dominant and nondominant sides in females (**F**)

For the discharge rate of the 60% MVC task, males did not show a significant side $\times$ MU_subgroup interaction (*F* = 1.182, *p* = 0.3191, *η*^2^ = 0.065) (Fig. 4A). There was a main effect of MU_subgroup (*F* = 7.294, *p* = 0.002, *η*^2^ = 0.302), whereby there was a significantly higher value of discharge rate in MU20 and MU40 than in MU60 (*p* = 0.006, 95% CI = − 10.754 to − 1.680 pps, and *p* = 0.032, 95% CI = − 8.245 to –0.324 pps) (Fig. 4B). The nondominant side showed a significantly higher discharge rate than the dominant side (*F* = 12.05, *p* = 0.0001, *η*^2^ = 0.2616721) (Fig. 4C). Females did not show a significant side $\times$ MU_subgroup interaction (*F* = 0.1812, *p* = 0.8351, *η*^2^ = 0.0112) (Fig. 4D), nor did they show a significant difference between sides (*F* = 0.9320, *p* = 0.2928, *η*^2^ = 0.028) (Fig. 4F). On the other hand, there was a main effect of MU_subgroup (*F* = 3.901, *p* = 0.0305, *η*^2^ = 0.196), whereby there was a significantly higher value of MU20 than MU60 (*p* = 0.015, 95% CI = − 9.953 to − 0.927) (Fig. 4E).

**Fig. 5:**
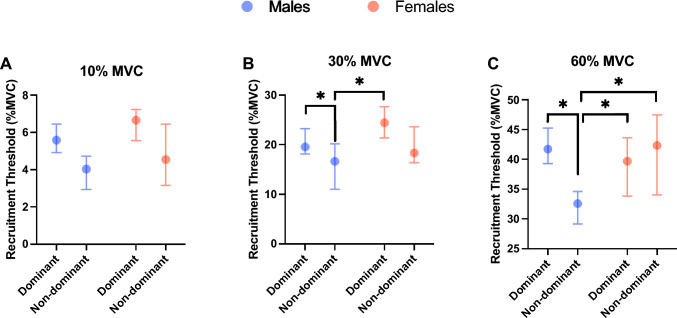
Comparison of recruitment thresholds between the dominant side and nondominant side in females and males at 10% (**A**), 30% (**B**), and 60% MVC (**C**). Males’ nondominant side showed a significantly lower recruitment threshold than females’ dominant side at 30% and 60% MVC and a significantly lower recruitment threshold than females’ nondominant side at 60% MVC. Furthermore, males’ dominant side showed a significantly higher recruitment threshold than their nondominant side at 30% and 60% MVC. Data are shown as the median and 95% CI. * *p* < 0.05

The recruitment threshold showed a significant sex $\times$ side $\times$ MVC interaction (*F* = 6.90, *p* < 0.0001, *η*^2^ = 0.094). The recruitment threshold was significantly higher on the dominant side than on the nondominant side at 30% and 60% MVC in males (*p* < 0.0001, 95% CI = 1.275 to 7.355% MVC and *p* < 0.0001, 95% CI = 6.356 to 12.742% MVC) but not in females (Fig. 5). At 30% MVC, males’ nondominant side showed a significantly lower recruitment threshold than females’ dominant side (*p* = 0.02 and 95% CI = 0.410 to 11.99% MVC). At 60% MVC, males’ nondominant side showed a significantly lower recruitment threshold than females’ nondominant side and dominant sides (*p* = 0.008, 95% CI = 0.940 to 14.114% MVC and *p* < 0.0001, 95% CI = 2.121 to 13.601% MVC, respectively).

The CV of force did not show a significant sex $\times$ side $\times$ MVC interaction (*F* = 1.70, *p* = 0.1835, *η*^2^ = 0.025) or a main effect of side (*F* = 0.32, *p* = 0.5712, *η*^2^ = 0.005) or MVC (*F* = 2.335, *p* = 0.0938, *η*^2^ = 0.034). On the other hand, there was a significant main effect of sex (*F* = 4.73,* p* = 0.0297, *η*^2^ = 0.067).

There was no significant sex $\times$ side interaction effect on the MVC (*F* = 0.007, *p* = 0.9334, *η*^2^ = 0.001) and no main effect of sex (*F* = 0.072, *p* = 0.7893, *η*^2^ = 0.002) and side (*F* = 0.2668, *p* = 0.6084, *η*^2^ = 0.007). Furthermore, there was no correlation between CSA and MVC in either males (*r* = − 0.2925, *p* = 0.2108) or females (*r* = 0.04766, *p* = 0.8250).

**Fig. 6:**
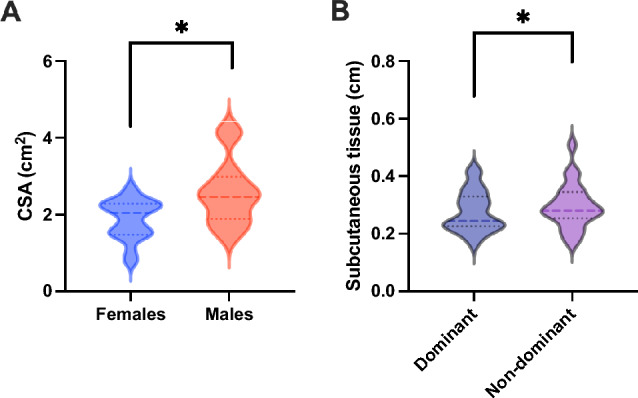
Comparison of muscle cross-sectional area between females and males (**A**) and subcutaneous tissue between the dominant side and nondominant side (**B**). Females showed a significantly lower cross-sectional area than males. The dominant side showed significantly less subcutaneous tissue than the nondominant side

There was no significant sex $\times$ side interaction effect on the CSA and subcutaneous tissue (*F* = 1.17, *p* = 0.2801, *η*^2^ = 0.052 and *F* = 2.95, *p* = 0.086, *η*^2^ = 0.123). There was a main effect of sex (*F* = 9.63, *p* = 0.0019, *η*^2^ = 0.314) on the CSA–males had a significantly higher CSA than females (95% CI = 0.326 to 1.363)–but not a main effect of side (*F* = 2.39, *p* = 0.200, *η*^2^ = 0.102, 95% CI =  − 0.720 to 0.155 cm^2^) (Fig. 6A). Furthermore, there was a main effect of side (*F* = 4.28, *p* = 0.0386, *η*^2^ = 0.169) on subcutaneous tissue, with the dominant side having significantly less subcutaneous tissue than the nondominant side (95% CI = 0.002 to 0.081 cm) (Fig. 6B).

## Discussion

This study compared sex-specific dominance-specific MU firing behavior in young adults using HD-sEMG. We found that males showed (1) significant asymmetry in discharge rates and recruitment thresholds according to dominance at moderate and high force output and (2) a significantly lower discharge rate and recruitment threshold than females. On the other hand, females did not show significant asymmetry in the above outcomes at each investigated force output. These results support our hypothesis that females and males have different MU control strategy characteristics.

We found that females exhibited a significantly higher discharge rate and recruitment threshold than males. In previous studies using HD-sEMG, females exhibited a higher discharge rate than males in various muscles (i.e., the VL muscle (Guo et al. 2022) and TA muscle (Taylor et al. 2022)). Furthermore, several previous studies reported that females showed a significantly higher recruitment threshold than males in the TA muscle (Martinez-Valdes et al. 2020) and VL muscle (Boccia et al. 2019). These studies were not strictly sex-specific, as there were also sex differences in muscle strength, and the effects of muscle fiber type and other factors must be considered. This study found sex differences in discharge rate and recruitment thresholds even when sex differences were accounted for in a muscle strength-matched population. A recent study using intramuscular EMG reported that sex differences in the properties of MUs in the TA muscle were more apparent when the MVC values were matched (Inglis and Gabriel 2020). Furthermore, Herda et al. also reported sex differences in MU firing behavior (discharge rate and recruitment threshold) in a muscle-matched subject group (8–10 years old) for the FDI muscle, as in the present study (Herda et al. 2019). Their findings support our results indicating sex differences in potential MU characteristics, including the discharge rate and recruitment threshold, in strength-matched subjects. The sex differences in MU firing behavior may be due to sex differences in corticospinal tract excitability associated with differences in brain anatomy, such as gray matter and white matter (Hanlon and McCalley 2022). Interestingly, previous studies reported that males are better at visual motor tracking tasks than females (Carey et al. 1994; Mathew et al. 2020). Anatomical and functional sex differences in the cerebellum (Raz et al. 2001), a structure important in eye-hand coordination (Miall et al. 2001), may be responsible for the sex differences in the visual coordination task. The results of our study also found that the CV of force in females was significantly higher than that in males. Similarly, a previous study reported higher values of CV of force and CV of the ISI in females than in males (Inglis and Gabriel 2021b). The CV of force has also been reported to affect MU firing behavior (Jakobi et al. 2018), and it is likely that the difference in eye-hand coordination while performing the motor task was also a factor in the sex differences in MU firing behavior of the FDI muscle. Hormonal influences have been implicated in these sex differences in the characteristics of MU firing behavior. The female sex hormone progesterone is known to affect neurotransmitter function (Callachan et al. 1987), and it has been reported that the MU discharge rate is higher after ovulation when the progesterone level is high (Tenan et al. 2013). On the other hand, a recent study reported that higher testosterone levels were associated with reduced MU action potential complexity (Guo et al. 2022). These findings indicate that sex hormones influence MU firing behavior and may be one of the reasons for the observed sex differences in MU characteristics. However, this study did not investigate hormonal dynamics. Future studies should clarify this point by analyzing the association of MU firing behavior with the circadian rhythm of hormones and the ovulatory cycle.

In this study, we found that females did not exhibit laterality in firing behavior compared to males. Lateral dominance of brain activity suggests functional specialization in either the left or right cerebral hemisphere (Steenhuis and Bryden 1989; Hebbal and Mysorekar 2006), resulting in the preferential use of the dominant limb to manipulate objects or initiate a movement (Peters 1988). Accordingly, an association between the characteristics of MU firing behavior and the dominant side has been well established (Kamen et al. 1992; Schmied et al. 1994; Adam et al. 1998). Specifically, the dominant hand has been reported to have lower discharge rates and recruitment thresholds than the nondominant hand in the FDI muscle as recorded with intramuscular EMG (Adam et al. 1998). These findings are consistent with the results of this study that the dominant side exhibited a lower discharge rate and lower recruitment threshold compared with the nondominant side during the isometric contraction task in males. On the other hand, females did not show a significant difference between the dominant and nondominant sides. This finding is consistent with the assumption of reduced asymmetrical organization in females (Shaywitz et al. 1995; Hausmann and Güntürkün 1999), possibly due to sex differences in cerebral function. FCA is thought to be generated by interhemispheric inhibition of the nondominant hemisphere by the dominant hemisphere. Several studies that identified sex differences have found that FCA is more pronounced in males than in females (Hausmann et al. 1998; Hausmann and Güntürkün 1999). Many researchers have reported sex differences in brain structure and function (Allen et al. 1991; Ingalhalikar et al. 2014; Björnholm et al. 2017). In adulthood, males exhibited higher fractional anisotropy (FA) and lower mean diffusivity than females in many regions (Westerhausen et al. 2003; Hsu et al. 2008; Abe et al. 2010; Ritchie et al. 2018). In contrast, females may have higher FA than males in parts of the corpus callosum (Schmithorst et al. 2008; Kanaan et al. 2014). FA is related to axonal packing and myelination (Beaulieu 2002). The corpus callosum is the region responsible for neurotransmission between the left and right cerebral hemispheres (Wahl and Ziemann 2008). Differences in neural networks in this region may lead to sex differences in information transfer between the hemispheres and influence asymmetries in motor nerve function between the dominant and nondominant hands.

The results of this study showed that CSA was significantly higher in males than in females, but there was not a significant sex difference in muscle strength, although there was a relative difference of 60%.

Previous studies have reported that males have higher CSA and muscle strength in the FDI muscle compared to females (Sars et al. 2018; Parra et al. 2020), but our results were not consistent with these studies. We analyzed the relationship between CSA and muscle strength in the FDI muscle and found no significant correlation between CSA and MVC of the FDI muscle. According to this finding, the muscle strength of the FDI muscle is influenced more by neuromuscular factors than by muscle mass. Although the subjects were different (amyotrophic lateral sclerosis), Jenkins et al. reported no correlation between muscle weakness and changes in CSA in the FDI muscle (Jenkins et al. 2013). This finding supports our hypothesis that muscle strength in the FDI muscle is influenced more by neural factors than by muscle mass. Furthermore, the possibility that the menstrual cycle affects muscle strength must be considered. Several studies on the effects of the menstrual cycle on muscle strength found no effects in the lower limb muscles (Kubo et al. 2009; Romero-Moraleda et al. 2019), but an effect has been reported for the hand muscles (Phillips et al. 1996). Our study and other studies targeting the FDI muscle (Sars et al. 2018; Parra et al. 2020) did not clearly account for menstrual cycles, which may have contributed to the variability in these results. Therefore, we believe that further studies addressing the menstrual cycle are needed in future investigations.

This study has several limitations. First, although both right- and left-handed individuals were recruited, the percentage of left-handed individuals was negligible for both females and males. A previous study noted that right- and left-handed individuals have different nerve conduction velocities (Patel and Mehta 2012); thus, it is desirable to analyze right- and left-handed individuals separately. Second, this study recruited only young adults. MU firing behavior changes with age (Watanabe et al. 2016), and there are sex differences in these changes (Piasecki et al. 2021). Therefore, the results may not generalize to older adults. Third, this study only included tasks up to 60% MVC. Previous studies reported that the majority of MUs in the FDI muscle are recruited prior to 50% MVC, with a few MUs recruited up to 70% MVC (Thomas et al. 1986; Kamen et al. 1995). De Luca et al. reported an increase in the MU firing rate in the FDI muscle up to 80% MVC with increasing torque exerted (De Luca et al. 1982). These findings indicate that MU yield decreases with increasing exercise intensity, and data at exercise intensities up to 80% MVC may provide information for further motor control. Fourth, we only performed HD-sEMG and did not directly assess whether there were sex differences in brain function; therefore, we can only speculate about neural network asymmetries during motor tasks. In the future, simultaneous measurements with HD-sEMG and electroencephalogram (EEG) should be performed to provide more detailed data on asymmetry of brain function through analysis of EEG signals and MU activity during motor tasks. Finally, only subjects with similar muscle strength and subcutaneous tissue thickness were included in this study. There are several factors known to influence EMG recordings, such as muscle strength, subcutaneous tissue thickness, and muscle size; thus, we cannot conclude that there is a causal relationship between those factors and the behavior of MUs according to HD-sEMG based on this study alone. In future studies that include a wide range of subjects, these relationships can be clarified to enable better interpretation of the HD-sEMG results. Finally, this study examined only the FDI muscle. A recent study analyzing MUs in the TA muscle reported that no asymmetry of MUs was observed in males (Petrovic et al. 2022). Since the upper and lower extremities are used differently, it is likely that the motor control mechanisms are also different, but sex differences in other muscles need to be clarified.

## Conclusions

We identified sex-specific laterality of MU firing behavior in young adults. Females exhibited higher discharge rates and MU recruitment thresholds than males. Furthermore, there was asymmetry in MU firing behavior in males, whereas no asymmetry was observed in females. Sex differences have been observed not only in motor function but also in disease severity, and clarification of neurophysiological sex differences in healthy individuals is important for rehabilitation medicine as well as for sports science. In the future, further details on sex differences in MUs can be elucidated by investigating the relationships of MUs with sex hormone dynamics and aging effects.

## Data Availability

Data are available from the corresponding author upon reasonable request.
